# Impact of hypertension diagnosis on morbidity and mortality: a retrospective cohort study in primary care

**DOI:** 10.1186/s12875-023-02036-2

**Published:** 2023-03-23

**Authors:** Jesus Martín-Fernández, Tamara Alonso-Safont, Elena Polentinos-Castro, Maria Dolores Esteban-Vasallo, Gloria Ariza-Cardiel, Mª Isabel González-Anglada, Luis Sánchez-Perruca, Gemma Rodríguez-Martínez, Rafael Rotaeche-del-Campo, Amaia Bilbao-González

**Affiliations:** 1 Oeste Family and Community Care Teaching Unit, Primary Care Assistance Management, Madrid Health Service, Madrid, Spain; 2 Department of Medical Specialties and Public Health, Faculty of Health Sciences, Rey Juan Carlos University, Madrid, Spain; 3 Health Services Research Network in Chronic Diseases, REDISSEC- ISCIII, Madrid, Spain; 4 Research Network On Chronicity, Primary Care and Health Promotion-RICAPPS (RICORS), ISCIII, Madrid, Spain; 5 Gregorio Marañón Health Research Institute (IISGM), Madrid, Spain; 6 Technical Directorate of Health Information Systems, Primary Care Assistance Management, Madrid Health Service, Madrid, Spain; 7 Research Unit, Primary Care Assistance Management, Madrid Health Service, Madrid, Spain; 8 Health Reports and Studies Service, General Directorate of Public Health, Ministry of Health, Madrid, Spain; 9 Internal Medicine Service, Alcorcón Foundation University Hospital, Madrid, Spain; 10 Don Luis Infant Health Center, Primary Care Assistance Management, Madrid Health Service, Madrid, Spain; 11 Alza Health Center, Osakidetza, OSI Donostia, Research Group in AP IIS Biodonostia, San Sebastián, Spain; 12 Osakidetza, Basque Health Service, Basurto University Hospital, Research and Innovation Unit, Bilbao, Spain; 13 Kronikgune Health Services Research Institute, Barakaldo, Spain; 14 Department of Medicine, Faculty of Health Sciences, University of Deusto, Bilbao, Spain

**Keywords:** Hypertension, Survival, Cardiovascular disease, Primary health care

## Abstract

**Background:**

Hypertension is responsible for a huge burden of disease. The aim of this study was to evaluate the impact of newly diagnosed hypertension on the occurrence of kidney or cardiovascular events (K/CVEs) and on mortality among community dwellers.

**Methods:**

Retrospective cohort study, conducted from January, 2007, to December, 2018. All patients (age > 18) newly diagnosed with hypertension and no previous K/CVEs in 2007 and 2008, in the primary care centers of Madrid (Spain) (*n* = 71,770), were enrolled. The control group (*n* = 72,946) included patients without hypertension, matched by center, sex and age. The occurrence of kidney or CV events, including mortality from these causes and total mortality were evaluated using Cox regression and multistate models. Data were collected from three sources: personal data from administrative records, clinical data from medical records, and mortality data from regional and national databases.

**Results:**

The median follow-up was 138.61 months (IQR: 124.68–143.97 months). There were 32,896 K/CVEs (including 3,669 deaths from these causes) and 12,999 deaths from other causes. Adjusted for sex, smoking, diabetes and socioeconomic status, K/CVEs HR was 4.36 (95% CI: 3.80–5.00) for diagnoses before 45 years of age, 2.45(95% CI: 2.28- 2.63) for diagnosis between 45 to 54 years, and HR decreased to 1.86 (95% CI: 1.64–210) for diagnoses over age 85. Total mortality risk was only higher for hypertension diagnosed before 55 years of age (HR: 2.47, 95% CI: 1.90–3.19 for ages 18 to 44; and HR: 1.14, 95% CI: 1.02–1.28 for ages 45 to 54).

**Conclusion:**

The diagnosis of hypertension in the community environment, in patients without evidence of previous kidney or CV disease, is associated with a large increase in the risk of K/CVEs, but especially in individuals diagnosed before the age of 55. This diagnosis is only associated with an increase in kidney or cardiovascular mortality or overall mortality when it occurs before age 55.

**Supplementary Information:**

The online version contains supplementary material available at 10.1186/s12875-023-02036-2.

## Background

Arterial hypertension (HTN) is one of the most prevalent pathological conditions. One in three people over age 30 has been diagnosed with HTN, and although the age-adjusted prevalence has remained stable, the total number of diagnoses has doubled in the past 30 years [1].

HTN is an enormous burden responsible for the loss of 143 million disability-adjusted life years (DALYs) worldwide by 2015, considering the threshold of 140/90 mm Hg for its definition. These figures represented an increase of more than 30% of the DALYs lost for the same reason in 1990 [2].

The prevalence of HTN increases throughout life; among a cohort of previously healthy patients ages 25, 45, and 65, 0.3%, 6.5%, and 37% were diagnosed, respectively [3]. The number of people over 65 is growing steadily. Specifically, in Europe, it is expected that their number will double during the next 50 years, reaching 150 million people, and those who reach the average life expectancy without HTN have more than a 90% probability of developing the disease during their remaining life [4].

The excess mortality produced by HTN is mainly mediated by CV disease [2, 5]. Although the control of HTN through pharmacological and lifestyle measures has been shown to decrease mortality from these causes [6–10], it seems that hypertensive patients have an excess risk of CVEs [11] and overall mortality [12–14]. There has been a reduction in mortality in hypertensive patients over time, but there is a differential mortality compared to nonhypertensive patients with the same characteristics [15].

However, some authors have questioned this interpretation [16]. The diagnosis of hypertension occurs more frequently in people with other cardiovascular risk factors (CVRFs) [17]. Further, the presence of certain inflammatory markers, which have been associated with cardiovascular disease, is associated with the risk of being diagnosed with hypertension [18]. In fact, the existence of other CVRFs in patients newly diagnosed with hypertension is more frequent than in the general population of the same age [19]. Although the association between hypertension and cardiovascular disease, and mortality seems solid, the causality is debatable.

The impact of hypertension on overall mortality is attenuated as a function of the age of onset, going from mortality risks 2.5 times higher when hypertension is diagnosed before the age of 45 to excess mortality risk lower than 30% when this diagnosis is made over 65 years [19]. It is possible that the increase in blood pressure levels with age may constitute a protective mechanism against the dysfunction of certain organs [20, 21]. Additionally, it has been described that the differences in mortality (by cardiovascular or global cause) between patients with and without hypertension disappear when only the group of patients with treated and good control is considered [22].

In this context, the objective of evaluating the impact of newly diagnosed hypertension on the occurrence of kidney or cardiovascular events, mortality from these causes and total mortality in different age groups in the community under clinical practice conditions was proposed.

## Method

A retrospective cohort study was designed.

Inclusion criteria for the hypertensive cohort included to be over 18 years of age at recruitment, have been diagnosed of hypertension (code CIAP2 K86) from January 1, 2007, to December 31, 2008, and the absence of kidney or cardiovascular disease prior to such diagnosis. The diagnosis of hypertension implied that the mean of two or more correctly measured systolic blood pressure readings at each of two or more clinic visits was ≥ 140 mmHg or that diastolic blood pressure readings at each of two or more clinic visits was ≥ 90 mmHg. When the record referred to a diagnosis prior to that time, the subject was excluded.

The comparison cohort was constructed by pairing each individual with another person from the same Primary Care Center (PCC), without hypertension of the same sex and age range who did not have kidney or cardiovascular (CV) disease.

Exclusion criteria were being younger than 18 years, having suffered a kidney disease or CV event or having been diagnosed with hypertension before the start of the study.

Subjects in each cohort were selected from all PCC in the Community of Madrid.

Additional file 1 details the construction of both cohorts.

The follow-up lasted until December 31, 2018, or until the patient was removed from the community health records or died.

Sociodemographic and clinical variables were collected.

Age in years at diagnosis, sex and deprivation index of the area at the time of inclusion in the study were recorded. This deprivation index was developed for the MEDEA Project using Principal Component Analysis with the Census data. MEDEA index detects small areas of large cities with unfavorable socioeconomic characteristics and is related to general mortality [23]. The index was assigned to each census area using the following five socioeconomic indicators: manual workers, unemployment, temporary wage earners, total insufficient education and in youth. Each patient was assigned the MEDEA Index (in quintiles, the fifth quintile represents the least advantaged group) of their PCC, as an approximation to the place of residence.

The presence of the following clinical conditions recorded in the Clinical History of Primary Care, which uses the International Classification of Primary Care (ICPC-2), was collected [24]:

Diabetes Mellitus (DM)-ICPC2 T89 and T90-, tobacco use -ICPC2 P17-, or any reference to active tobacco consumption in the Electronic Health Record (HER) at the time of inclusion or in the year prior to inclusion.

In the follow-up, three types of dependent variables were collected:

- Occurrence of kidney or cardiovascular event (K/CVE): ischemic heart disease (acute myocardial infarction (ICPC2 K75), angina–(ICPC2 K74), chronic ischemia (ICPC2 K76), heart failure (ICPC2 K77), cerebrovascular disease (ICPC2 K90, K91), peripheral arterial disease (ICPC2 K92), chronic kidney disease (ICPC2 U99.1), or appearance of maintained urinary microalbuminuria, or proteinuria.
- Mortality from all causes. The International Classification of Diseases 10th edition (ICD-10) was used to study the causes of mortality [25].
- Kidney or CV mortality: deaths due to chronic kidney disease (ICD10: N18), cerebrovascular accident (ICD10: G46; I60-I69), ischaemic heart disease (ICD10: I20-I25), heart failure (ICD10: I50) and peripheral arterial disease (ICD10: I70, I71, I72, I74), were classified as kidney or CV mortality.

The appearance of hypertension in the cohort that initially did not express this condition was also collected.

### Data sources

Potential participants were identified by applying the eligibility criteria to the Center for Basic Strategic Information for Health care Environments (CIBELES). Clinical data were collected using a coding algorithm from the Centralized EHR for Primary Care PC of the Community of Madrid (AP-Madrid®). The EHR electronic source was linked to the mortality database of the Statistics National Institute and copied to a normalized database.

### Analysis

Prior to analysis, investigators implemented and verified several data quality processes for error identification and had access to the database population.

For the study of the occurrence of events, it should be taken into account that the subjects of the unexposed cohort could be diagnosed with hypertension in the follow-up. To use time-dependent covariates, the observation periods must be broken down into parts, depending on whether there is exposure. Once this procedure is performed, the data can be analyzed using a Cox proportional hazards model [26]. Given that contextual data were used, standard errors were calculated using robust methods and adjusted for 401 clusters (centers) [27]. The first analytical approach was Cox regression with time-dependent covariates.

On the other hand, mortality from kidney or CV causes and mortality from other causes can be considered competitive risks. One of the events could increase by decreasing the other. To address this problem, multistate models were constructed [28]. These models assume that the probability of transitioning to another state only depends on the present situation and allows treating some competitive risks as mutually exclusive absorbing states. Each transition between the states can be evaluated by Cox regressions, and the probabilities of transition to the same state from two different intermediate states can be compared [26].

**Fig. 1:**
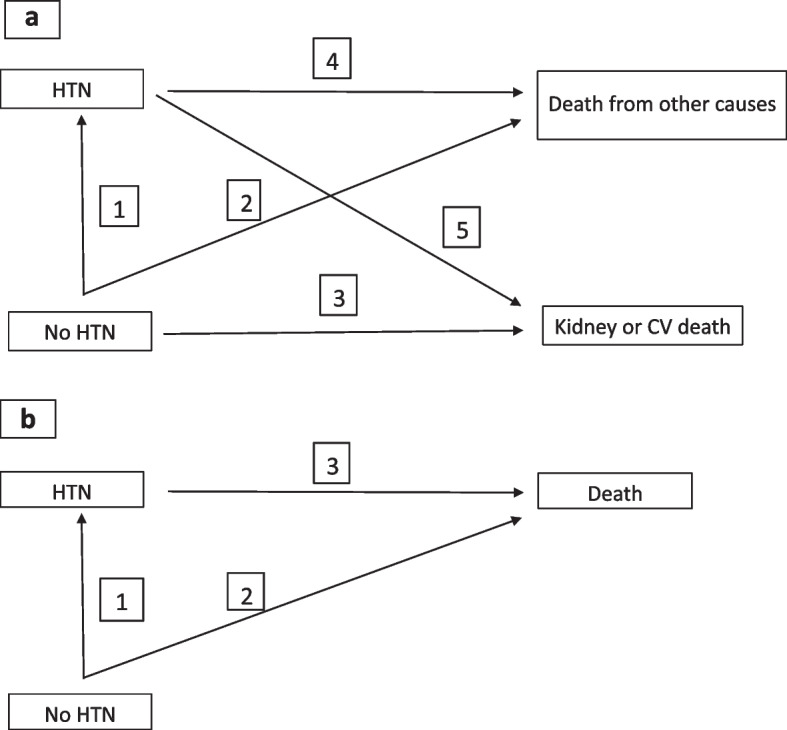
Transitions evaluated using multistate models. **a** Mortality from different causes. **b** Total mortality

The transitions defined for the multistate models can be seen in Fig. 1a and b. In our case, the comparisons of interest were transitions 2 versus 4 and 3 versus 5 to assess the risk of death from other causes and death from kidney or CV causes, respectively (Fig. 1a). To assess the impact of the association between HTN and total mortality, transitions 2 and 3 of Fig. 1b were studied. The comparison between models was made by assessing the Akaike Information Criteria (AIC) and Bayes Information Criteria (BIC) [29]. The analyses were performed with Stata® 14 using the “multistate” module designed by Crowther and Lambert [30].

### Ethical and legal aspects

A positive opinion was obtained from the Ethical Committee of the Alcorcon Foundation University Hospital.

This study was funded by the Carlos III Health Institute (ISCIII) through project PI18/00370 and co-financed by the European Union.

## Results

A total of 71,770 patients with an incidental diagnosis of hypertension and 72,946 matched controls by age group, sex and health center were included in the study (see Additional file 1).

Table 1 shows the baseline characteristics of both cohorts.

**Table 1:** Characteristics of the selected cohorts

	Hypertensive cohort	Not hypertensive at baseline
Total	71.770	72.946
Ages		
18 to 44 years	19.06%	19.13%
45 to 54 years	24.30%	24.29%
55 to 64 years	26.33%	26.33%
65 to 74 years	18.70%	18.63%
75 to 84 years	9.54%	9.53%
85 and older	2.07%	2.09%
Women	51.76%	51.78%
Smokers	16.39%	18.05%
Diabetes mellitus	10.15%	3.97%

The median follow-up was 138.61 months (interquartile range, IQR 124.68–143.97 months).

During the follow-up time, 15,042 patients in the unexposed cohort were diagnosed with hypertension. Of these, 1,327 had suffered a K/CVE prior to the diagnosis of hypertension, so their observation period ended at that time (with the occurrence of K/CVE). The remaining 13,715 were considered subjects with hypertension.

### Study of the occurrence of kidney or CV events 

A total of 32,896 K/CVEs were recorded, including death due to these causes, 13,008 in the initially unexposed cohort (17.83%; 95% CI: 17.55–18.11%) and 19,888 (27.71%; 95% CI: 27.38–28.03%) in the cohort diagnosed with hypertension.

For patients who had a K/CVE, the median follow-up until the event was 70.14 months (IQR: 39.79–100.86 months). The follow-up was performed on 144,716 subjects, with a total of 16,462,184.38 person-months of observation and an event incidence rate of 0.0020 per person-month.

The risk of event occurrence was studied for each age range (constructing an age-hypertension interaction) and adjusting it for the following covariates: sex, smoking, diabetes diagnosis and socioeconomic status of the area.

Table 2 shows the results of the best Cox model. The risk of event occurrence is up to four times higher when hypertension diagnosis is made between ages 18 and 44 and gradually decreases with increasing age of diagnosis, but the association is relevant in all age ranges.

**Table 2:** Cox model for cardiovascular events, including kidney or cardiovascular mortality, hypertensive vs. non-hypertensive subjects

Variable	HR	HR CI 95%	*p* value
Hypertension in each age group ^a^			
18 to 44 years	4.358	3.799- 4.999	< 0.001
45 to 54 years	2.445	2.275–2.628	< 0.001
55 to 64 years	1.962	1.870–2.060	< 0.001
65 to 74 years	1.919	1.836–2.005	< 0.001
75 to 84 years	1.718	1.629–1.812	< 0.001
85 and older	1.856	1.644–2.096	< 0.001
Female vs. male	0.866	0.844–0.889	< 0.001
Diabetes mellitus	1.494	1.443–1.547	< 0.001
Baseline smoking	1.311	1.274–1.349	< 0.001
Socioeconomic group			< 0.001
2nd vs. 1st quintile	1.072	0.997–1.153	0.061
3rd vs. 1st quintile	1.169	1.083- 1.263	< 0.001
4th vs. 1st quintile	1.178	1.101–1.261	< 0.001
5th vs. 1st quintile	1.224	1.128–1.328	< 0.001
Characteristics of the model	Akaike Information Criteria (AIC): 739,153.3 Bayes Information Criteria (BIC): 739,332.7		

^a^ The HR (hazard ratio) expresses the risk of an event calculated from the linear combination of the hypertension coefficients and their interaction with age

### Study of global mortality

The follow-up was performed on 144,716 subjects, with a total of 18,137,117.42 person-months of observation and an incidence rate of death of 0.0009 per person-month. At the end of the follow-up, 16,668 subjects had died, 10.74% (95% CI: 10.51–10.97%) of the cohort initially diagnosed with hypertension and 12.28% (95% CI: 12.05- 12.52%) of the cohort initially undiagnosed. Of the deaths observed, 3,669 were caused by kidney or CV events and 12,999 by other causes.

For patients who died, the median follow-up was 84.40 months (IQR: 52.53–112.21 months).

The overall mortality risk was studied for each age range (constructing an age-hypertension interaction) and adjusting it for the same variables as in the previous case.

Table 3 shows the results of the best model. Adjusted for sex, smoking, diabetes and the socioeconomic level of the area, hypertension increases the risk of mortality up to 54 years but stops doing so when diagnosed after this age.

**Table 3:** Cox model for the total mortality event, hypertensive vs. non-hypertensive subjects

Variable	HR	HR CI 95%	*p* value
Hypertension in each age group^a^			
18 to 44 years	2.465	1.904–3.192	< 0.001
45 to 54 years	1.141	1.017–1.281	0.025
55 to 64 years	0.864	0.803- 0.932	< 0.001
65 to 74 years	0.794	0.742–0.850	< 0.001
75 to 84 years	0.738	0.696- 0.782	< 0.001
85 and older	0.997	0.907–1.095	0.950
Female vs. male	0.618	0.597–0.640	< 0.001
Diabetes mellitus	1.243	1.179–1.310	< 0.001
Baseline smoking	1.458	1.391–1.528	< 0.001
Socioeconomic group			0.041
2nd vs. 1st quintile	1.081	1.007–1.159	0.030
3rd vs. 1st quintile	1.018	0.949–1.042	0.618
4th vs. 1st quintile	0.994	0.933–1.060	0.872
5th vs. 1st quintile	1.055	0.988–1.128	0.108
Characteristics of the model	Akaike Information Criteria (AIC): 357,599.3 Bayes Information Criteria (BIC): 357,778.8		

^a^The HR (hazard ratio) expresses the risk of an event calculated from the linear combination of the hypertension coefficients and their interaction with age

### Study of mortality from different causes as competitive risks

A multistate model was developed in which the probability of mortality due to kidney or cardiovascular causes or other causes in the two cohorts was studied.

Table 4 shows the results of the association of hypertension with mortality in the different age groups. The adjustment variables were the same: sex, smoking, diabetes and socioeconomic status of the area.

**Table 4:** Proportional risks of death, hypertensive vs. non-hypertensive patients by age groups and causes (use of multistate models, Figs. 1a and b). Models adjusted for age, sex, diabetes mellitus, smoking and socioeconomic status of the area

Mortality due to kidney or cardiovascular causes			
Age group	HR	IC 95% HR	*p* value
18 to 44 years	9.309	3.819–22.694	< 0.001
45 to 54 years	1.787	1.280–2.493	0.001
55 to 64 years	1.045	0.855–1.278	0.665
65 to 74 years	0.959	0.828–1.111	0.578
75 to 84 years	0.900	0.811–0.999	0.049
85 and older	1.132	0.976–1.313	0.102
Characteristics of the model	Akaike Information Criteria (AIC): 75,448.71 Bayes Information Criteria (BIC): 75,628.22		
Mortality due to other causes			
Age group	HR	IC 95% HR	*p* value
18 to 44 years	2.044	1.557–2.683	< 0.001
45 to 54 years	1.082	0.959–1.222	0.200
55 to 64 years	0.870	0.801–0.945	< 0.001
65 to 74 years	0.814	0.757–0.875	< 0.001
75 to 84 years	0.750	0.701–0.802	< 0.001
85 and older	0.987	0.881–1.106	0.818
Characteristics of the model	Akaike Information Criteria (AIC): 280,556.7 Bayes Information Criteria (BIC): 280,736.2		
Total mortality			
Age group	HR	IC 95% HR	*p* value
18 to 44 years	2.466	1.905–3.193	< 0.001
45 to 54 years	1.154	1.028–1.295	0.016
55 to 64 years	0.891	0.827–0.960	0.003
65 to 74 years	0.837	0.783–0.895	< 0.001
75 to 84 years	0.785	0.740–0.833	< 0.001
85 and older	1.031	0.938–1.134	0.522
Characteristics of the model	Akaike Information Criteria (AIC): 356,480.7 Bayes Information Criteria (BIC): 356,660.2		

Hypertension is strongly associated with kidney or cardiovascular mortality when diagnosed in individuals less than 45 years of age, and this association remains but with lower intensity until 54 years of age. After this age, there is no association between the two. When other causes of mortality are evaluated, hypertension is associated with an increase in mortality only if it is diagnosed before age 45 and presents an inverse association after the age of 55.

## Discussion

The diagnosis of HTN in patients without previous kidney or CV disease is associated with an increase in the occurrence of K/CVEs (including death due to these causes) throughout the entire life course, but especially when HTN is diagnosed before the age of 55.

The diagnosis of hypertension is only associated with kidney or cardiovascular mortality or total mortality, when it occurs before 55 years. An inverse association has been observed between HTN diagnosis over 55 years and overall mortality. The described associations were found in patients without previous kidney or CV disease who were followed and treated in a health system with full access to the general population and adjusted for the effect of DM, smoking, and socioeconomic situation.

HTN is associated with an increase in CVEs [2, 22, 31] and an association has also been described between HTN and all-cause mortality [14]. In some studies, CVEs’ incidence was twice if HTN was diagnosed under the age of 45 and an the excess of risk was about 60% for patients diagnosed between 45 and 55 years [19]. The results presented indicate higher risks (HR 4.36 and 2.45 for each of these age ranges) but include kidney or CV death as an event. But association with mortality from all causes has only been found when HTN is diagnosed at the earliest ages of life. The differences are more subtle when we compare the results with studies that analyze newly diagnosed HTN by age strata. While the diagnosis of HTN has been associated with an excess risk of mortality from all causes of 2.5 times (HR 2.59) when it occurs before age 45, this excess mortality does not reach 30% (HR 1.29) when it occurs over age 65 [19]. In the age group under 45 years, our results are very similar for this association (HR 2.47). The decrease in risk with the latest diagnosis of HTN is consistent with what has been previously described. An increase in the probability of developing target organ injury has been reported in patients with HTN diagnosed before 35 years, which was not observed when the diagnosis was made over age 45 [32]. Some studies have reported an association between well-controlled HTN and all-cause mortality in patients younger than 70 years [33], but other ones reported no association observed with all-cause mortality in patients with HTN under treatment, older than 75 years [7].

The differences found when assessing the risk of total mortality may be due to several reasons. Some of the primary studies that mentioned cohorts were recruited more than two decades ago and those that have more recent recruitments find more uncertain results for the association of HTN with all-cause mortality [14]. Improved survival in HTN patients has been demonstrated over time, and when blood pressure levels are better controlled with antihypertensive medication [7, 9]. In one of the studies with the longest reported follow-up (median 19.1 years), although a strong association between HTN and the occurrence of CVEs and all-cause mortality for untreated or poorly controlled patients was established, no such association was found in treated and controlled patients [22].

The inverse association found between HTN and total mortality over 55 years should not be explained from a causal perspective, as it is not plausible. It has been reported that a more intense use of PC services was associated with lower mortality in hypertensive patients [34], and certain promotional interventions have been shown to decrease CV risk in elderly hypertensive patients in PC [35]. Additionally a strong association has been reported between higher continuity of care and reduced mortality rate among hypertensive patients [36]. Our health system has practically universal coverage. Chronic disease is the main explanatory factor for the use of family doctor visits [37], and HTN occupies a relevant consultation time in PC [38]. The health care to which hypertensive patients older than 55 years are subjected, as well as the action on other coexisting CVRFs, may contribute to explaining, at least in part, the association.

Regarding the confounding role of the variables studied, both the occurrence of kidney or CV events and mortality from all causes increased in diabetic patients and smokers. The role of these CVRFs in mortality is well known, and both factors are used as adjustment variables in most of the studies discussed [14, 31, 33, 39]. In women with HTN, the risk is lower. It has already been described that avoidable mortality is lower in women than in hypertensive men [40], and it has been estimated that the burden of disease for hypertension is lower in women than in men for all ages except over 75 years [2]. The association of a worse socioeconomic situation and the events associated with HTN has also been widely described [41, 42].

This study has limitations inherent to retrospective cohort studies. The strength of the data is determined by the quality of the information collected. Some of the classical CVRFs, such as hypercholesterolemia or obesity, or other clinical circumstances such as the time at which diabetes was diagnosed, were not included as adjustment variables because they had not been validated in the EHR or their collection could have been differential for the groups compared. The diagnoses recorded in the EHR of PC for HTN and DM have been previously validated [43].

Among the strengths, all cases diagnosed in PC in the Autonomous Community during a period of two years were included and the secondary data sources allowed us to identify the final state in a reliable way, with very limited losses to follow-up. Given the characteristics of the health system (in 2020, 86% of assigned people were visited in their PCC) the generalizability of the results is important.

The value of the findings presented is based on the fact that they are data in real clinical practice conditions, in a specific environment, in which paradoxically, although healthy lifestyle habits are not very prevalent, cardiovascular mortality remains comparatively low [44].

## Conclusion

The diagnosis of hypertension in the community environment, in patients without evidence of previous kidney or CV disease, is associated with a large increase in the risk of K/CVEs. This increased risk depends on the individual’s age at diagnosis; risk is highest if diagnosis is made before the age of 55 years and decreases with age. This diagnosis is only associated with an increase in kidney or cardiovascular or overall mortality when it occurs before age 55. Thus, the health system should increase secondary prevention measures, especially in hypertensive patients diagnosed before the age of 55, when excess risk is most evident.

## Supplementary Information

**Additional file 1:** Generation of Cohorts.

## Data Availability

The datasets used and analyzed during the current study are available from the corresponding author on reasonable request.

## Abbreviations

AIC: Akaike Information Criteria
BIC: Bayes Information Criteria
CIBELES: Center for Basic Strategic Information for Healthcare Environments
CV: Cardiovascular
CVE: Cardiovascular event
CVRF: Cardiovascular risk factor
DM: Diabetes Mellitus
DALY: Disability Adjusted Life Years
HTN: (Arterial) hypertension
ICD-10: International Classification of Diseases, 10th edition
ICPC2: International Classification of Primary Care 2nd ed.
K/CVEs: Kidney or cardiovascular events
MEDEA: Models and their Effects on Development paths an Ethnographic and comparative Approach (a method to examine mortality and socioeconomic and environmental inequalities in Spanish communities)
PC: Primary Care
PCC: Primary Care Center
