# Supplementary material for: Impact of hypertension diagnosis on morbidity and mortality: a retrospective cohort study in primary care
Source: BMC Prim Care. 2023 Mar 23;24:79. doi: 10.1186/s12875-023-02036-2 (PMC10037862; doi:10.1186/s12875-023-02036-2)
Supplement: Supplementary file 1 — Additional file 1: Generation of Cohorts. [file 12875_2023_2036_MOESM1_ESM.docx]

Appendix 1 Generation of Cohorts

Population> 18 years with "de novo" diagnosis of hypertension in 2007 and 2008 vs Random sampling stratified by health center, sex and age bracket

Exclusion >100 years at the date of inclusion, without annotation in EHR in the follow-up

Exclusion of duplicate patient identification codes

Exclusion of subjects with date of death prior to date of inclusion in the study

Review criterion exclusion treatment antihypertensive for error consideration dates

Exclusion of the cohort not exposed in a random and stratified way. No replacement

Exclusion of subjects without registration in CIBELES on 31/12/2008

Exclusion of subjects with date of registration of the diagnosis ≥ 30 days

Exclusion of cohort not exposed in a random and stratified way. No replacement

**Unexposed Cohort. N=85955**

**Exposed Cohort. N=85955**

N=85955

Excluded: 0

N=85909

Excluded: 46

N=85894

Excluded: 15

N=85673

Excluded: 221

N=84041

Excluded: 1632

N=83505

Excluded: 536

**N=71770**

Excluded: 11735

N=85943

Excluded: 12

N=85734

Excluded: 209

N=84266

Excluded: 1468

**N=72946**

Excluded: 11320

* EHR: Electronic Health Record; CIBELES: Center of strategic basic information for health environments
